# Postural behavior recognition of captive nocturnal animals based on deep learning: a case study of Bengal slow loris

**DOI:** 10.1038/s41598-022-11842-0

**Published:** 2022-05-11

**Authors:** Yujie Lei, Pengmei Dong, Yan Guan, Ying Xiang, Meng Xie, Jiong Mu, Yongzhao Wang, Qingyong Ni

**Affiliations:** 1 College of Information Engineering, Sichuan Agricultural University, Yaan, 625014 China; 2 Sichuan Key Laboratory of Agricultural Information Engineering, Yaan, 625000 China; 3 Key Laboratory of Livestock and Poultry Multi-omics, Ministry of Agriculture and Rural Affairs, College of Animal Science and Technology, Sichuan Agricultural University, Chengdu, 611130 China; 4 College of Life Science, Sichuan Agricultural University, Yaan, 625014 China

**Keywords:** Animal behaviour, Computational models, Image processing

## Abstract

The precise identification of postural behavior plays a crucial role in evaluation of animal welfare and captive management. Deep learning technology has been widely used in automatic behavior recognition of wild and domestic fauna species. The Asian slow loris is a group of small, nocturnal primates with a distinctive locomotion mode, and a large number of individuals were confiscated into captive settings due to illegal trade, making the species an ideal as a model for postural behavior monitoring. Captive animals may suffer from being housed in an inappropriate environment and may display abnormal behavior patterns. Traditional data collection methods are time-consuming and laborious, impeding efforts to improve lorises’ captive welfare and to develop effective reintroduction strategies. This study established the first human-labeled postural behavior dataset of slow lorises and used deep learning technology to recognize postural behavior based on object detection and semantic segmentation. The precision of the classification based on YOLOv5 reached 95.1%. The Dilated Residual Networks (DRN) feature extraction network showed the best performance in semantic segmentation, and the classification accuracy reached 95.2%. The results imply that computer automatic identification of postural behavior may offer advantages in assessing animal activity and can be applied to other nocturnal taxa.

Animal behavior can be generally defined as a decision-making process, which is a balance between a set of solutions that can guarantee high levels of welfare and allow animals to be independent of the surroundings and the environment^1^. The long-term repetition of stressful situations may lead to the repetition of the same associated postural behavior and make them become chronic states^2^. Thus, the measurement of animal activity and the related bio-processes and bio-responses are crucial in welfare assessment and captive management. Specifically, frequent monitoring of animals’ postural behavior in quantitative terms helps captive managers to verify the welfare state by early recognition of health anomalies evidenced through reduced locomotion, food intake, or social behaviors^3^, and the early detection of pathology symptoms, injuries, or problems in the captive environment^4^. As an initial step, the precise identification of these behaviors plays a vital role in ensuring unbiased monitoring results that affect decision-making. Video-based data collection is increasingly used in animal behavior monitoring^5^. Given that artificial recording and video analysis are both highly labor-intensive and possibly bias-prone, automatic behavior recognition could become critical to achieve acceptable throughput^6,7^. Advanced technologies like machine learning, deep learning, and artificial intelligence, as well as big data technologies and high-performance computing, have emerged in recent years and opened new modes for data-intensive research^8^.

Deep learning is a machine learning method based on artificial neural networks with representation learning, and it allows computational models with multiple layers to learn representations of data with multiple levels of abstraction^9^. As deep learning has been successfully applied into various domains of science, business and government, it has made major advances in animal studies^10^. Particularly, the convolutional neural network (CNN) has been extensively used in face and action recognition of wild and domestic fauna species, e.g. golden monkey (*Rhinopithecus roxellana*)^11^, giant panda (*Ailuropoda melanoleuca*)^12^, pig (*Sus scrofa domestica*)^13^, and Tibetan antelope (*Pantholops hodgsonii*)^14^. As one of the fundamental problems in deep learning, object detection is intended to find targeted objects in the images or videos and determine their categories and positions, representing the core issues of computer vision^15^. Thus it has been related to many applications including face recognition, behavior analysis and autonomous driving^16^. For images containing cluttered background and diverse object parts, however, object detection is not skilled in dealing with precise classification. As another research hotspot in deep learning, semantic segmentation divides an image into several parts based on similar characteristics and common pixel points and processes the image at the pixel level, and thus it can minimize negative background effects^17^. Therefore, the combined methods have been increasingly applied in the individual identification and action recognition of animals^13,18^.

Slow lorises (*Nycticebus* spp., Lorisidae) are small, arboreal and nocturnal primates native to south-east Asia^19^. All the species have been listed on Appendix I of the Convention on International Trade in Endangered Species of Wild Fauna and Flora (CITES). While the wild populations have dramatically declined due to habitat loss and hunting, a considerable number of individuals are illegally traded as pets, and confiscated into zoos and rescue centers^20,21^. Given the limited capability of the rescue facilities, captive lorises may suffer from incorrect diet, wounds or disease, and fear or distress^22,23^. In typical husbandry environments, it is unlikely that the welfare of slow lorises can be sufficiently addressed, and the levels of low welfare may be at the root of captive lorises’ abnormal behavioral patterns. A few studies have reported that a large proportion of confiscated individuals display stereotypies, appetitive behaviors and inappropriate social interactions^24,25^. Consequently, slow lorises may experience elevated mortality and perish quickly in captivity, making their reintroduction success impossible^22^.

Comparisons of activity patterns, particularly postural behavior under different ecological conditions, allow for exploration of behavioral ecology, conservation and captive management. Among the nocturnal primates, postural modes were categorized into an equally varied array including slow climbing, bridging, branch running and walking, and vertical clinging and leaping, and slow lorises are considered slow climbing specialists^26,27^. The “slow” locomotion mode and the large numbers of captive individuals make the slow loris an ideal model for behavior monitoring. Previous studies have also reported that their postural behavior is influenced by variation in their environment^28,29^. For captive or semi-captive wild animals, action recognition is crucial to make assessment of their welfare status and conduct best-practice reintroduction releases^30,31^. For instance, proficient locomotion skills are critical for the orangutans to safely and efficiently forage high up in trees^32^. Due to the large amount of humanpower and time that occurs in traditional observation and monitoring of nocturnal research^20^, however, obtaining precise, quantitative descriptions of postural behavior remains a challenge.

We established a human-labeled dataset for postural behavior recognition of captive Bengal slow lorises (*N. bengalensis*) and propose an object detection + semantic segmentation model. For the first time we introduce deep learning technology into automatic behavior identification of nocturnal primates based on a night-vision video system. The framework will contribute to researchers’ abilities to conduct high-throughput analysis of animal behavior in a short period of time, and enhance the possibilities for constant monitoring. Compared with other approaches, successfully established computer evaluation can offer the advantage of seamless data processing from real-time videos, without additional cost or personnel effort. Together with further machine learning techniques, automatic postural behavior recognition can be used to generate animal activity overviews and thus represent potential indicators for animal welfare, conservation and captive management.

## Method

### Definition of postural behavior

In this study, we aimed to validate the feasibility of computer vision in identifying the general behavior of captive slow lorises. Though the detailed ethograms have been provided in a few literatures^33,34^, we used a simplified postural behavior classification defined as follows:

*Feeding*: gnawing, biting, grabbing, licking, and chewing food.

*Moving*: body stretching and climbing.

*Resting*: staying at a certain position and keeping immobility.

*Socializing*: contacting or proximity (< 0.3 m) between individuals.

While *feeding* and *socializing* can be identified by the main parts of the bodies and the neighboring appendages (e.g. water and food bowls) or individuals, *moving* and *resting* cannot be precisely recognized due to similar image characteristics in object detection. Thus we combined the *moving* and *resting* behavior into *move-rest* in the object detection experiment, and identified them by semantic segmentation.

### Data collection

The data were collected from three wildlife rescue centers in Dehong, Xishuangbanna, and Puer, Yunnan, China. The Bengal slow lorises were housed together in a single cage in each site (Table 1). All the enclosures were simply enriched by dry wood and covered with iron wire mesh. The activities of slow lorises were constantly recorded by a night vision monitoring system (TCNC9401S3E-2MP-I5S and TC-NC9501S3E-2MP-I3S infrared camera, Tiandy Technologies CO., LTD., Tianjin, China). More than 100 TB video files were obtained from the surveillance cameras installed on the top of the cages from April 2017 to June 2018, with a resolution of 1920 × 1080 pixels. We extracted the frames at 2 s intervals and after excluding duplicate and similar pictures, and selected 1600 monitoring screenshots as the YOLOv5 object detection dataset. We marked the location of each individual whose action and postural behavior could be clearly identified, and classified into three postural behavior types: *feeding*, *move-rest* and *socializing*. After object detection using YOLOv5, we screened out 4,200 images referring to all the behavior types, and further screened out 1,000 images related to *move-rest* (containing *moving* and *resting*) for semantic segmentation. In the object detection and the semantic segmentation classification process, the dataset is divided into training set and test set at a ratio of 7:3.

**Table 1:** Enclosure characteristics of each captive site for video data collection.

Captive site	Dehong	Xishuangbanna	Puer
Coordinate	24.38287°N, 98.45872°E	22.39276°N, 100.89636°E	22.62198°N, 101.08916°E
Altitude (m)	850	1060	1600
Annual mean temperature (℃)	19.6	17.5	17.5
No. of individuals	4	9	9
No. of enclosures	1	1	1
Enclosure size (L × W × H) (m)	3.5 × 3.4 × 3.8	5.7 × 4.2 × 3.5	3.5 × 2.1 × 2.0
No. of cameras	2	3	2

### Experimental environment

All the experiments were conducted in the Sichuan Key Laboratory of Agricultural Information Engineering on a Lenovo Thinkstation P920 (Server number TSP920-C621). We build a network based on the Pytorch framework (Python version: 3.6.13; Torch version 1.8.0) under the Windows 10 system. The server used is configured with Inter Xeon Gold 5218 CPU, two NVIDIA Quadro RTX 5000 graphics cards, and 128G memory.

### Overall framework

**Figure 1:**
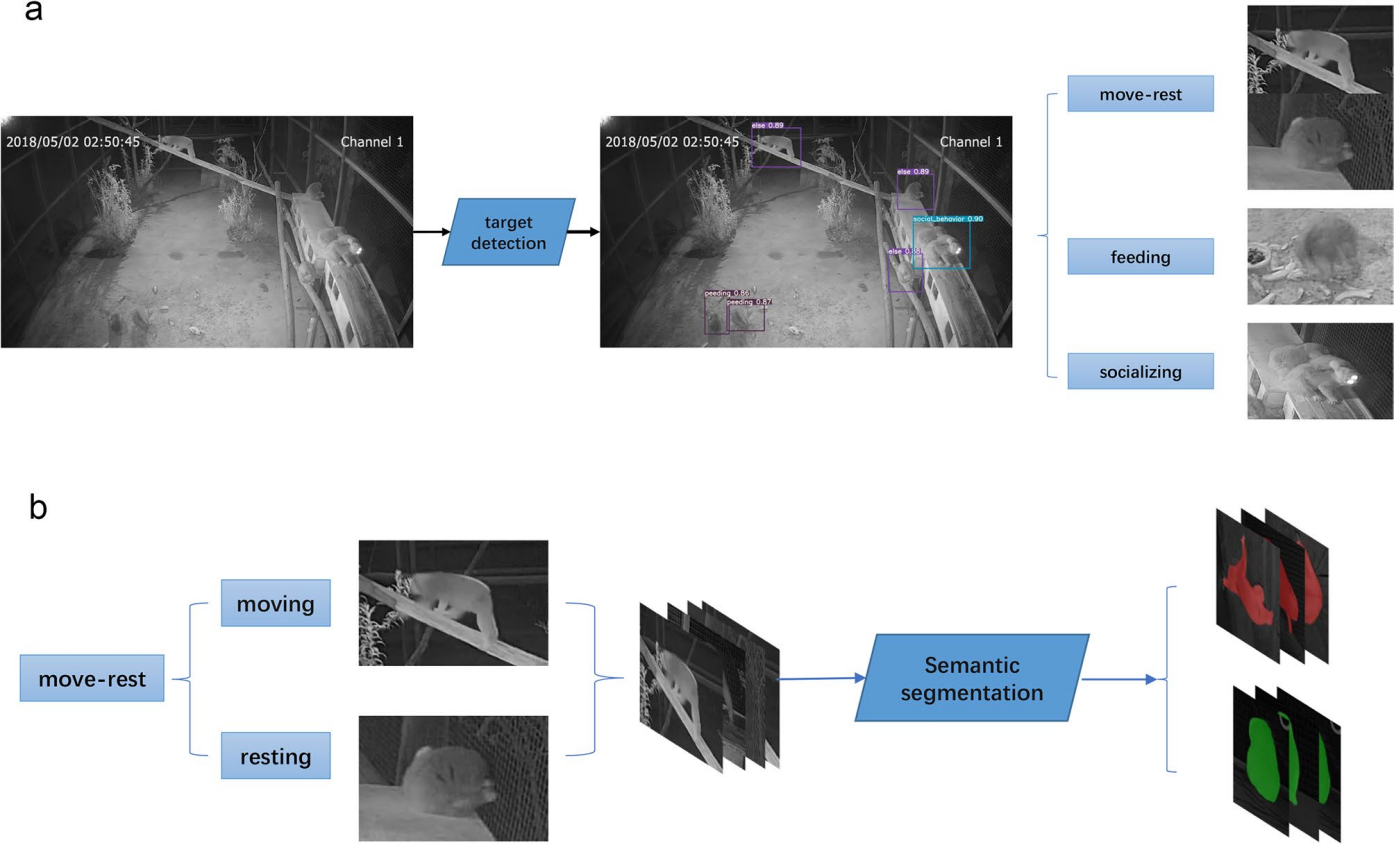
Processing of target detection, semantic segmentation and classification.

We annotated the postural behavior images collected from monitoring video files and divided them into three categories: *feeding*, *socializing* and *move*-*rest* (Fig. 1). The dataset obtained by YOLOv5 was labeled with Labelme, and then the DeepLabv3 + network was used to extract the contour of the loris individual and classify the behavior *move-rest* into *moving* and *resting*.

### Evaluation index

We used precision, recall, average Precision (AP), mean average precision (mAP), semantic segmentation accuracy (Acc), classification accuracy (Acc_class_) as evaluation criteria for the *i*-type. The definition is shown as follows:

$${\text{Precision}}_{{{\text{all}}}} = \frac{{\text{The number of postural behavior whose category is correctly predicted}}}{{\text{The number of postural behavior predicted in all categories}}}$$

$${\text{Precision}}_{i} = \frac{{{\text{The number of correctly predicted postural behavior in the }}i - {\text{th category}}}}{{{\text{The total number of postural behavior predicted to be the }}i - {\text{th category}}}}$$

$${\text{Recall}}_{i} = \frac{{{\text{The number of correctly predicted postural behavior in the }}i - {\text{th category}}}}{{{\text{The actual number of postural behavior detected as the }}i - {\text{th category}}}}.$$

$${\text{AP}}_{i} = \frac{{{\text{The sum of all precision of the the }}i - {\text{th postural behavior }}}}{{{\text{The number of all pictures with the }}i - {\text{th postural behavior}}}}$$

$${\text{mAP}} = \frac{{\text{The sum of the average accuracy of all categories}}}{{\text{The number of categories}}}$$

Acc is used to calculate the ratio between the number of correctly classified pixels and the total number of pixels. The parameters are defined in Table 2.

$${\text{Acc}} = \frac{{{\text{TP}} + {\text{TN}}}}{{{\text{TP}} + {\text{TN}} + {\text{FP}} + {\text{FN}}}}$$

**Table 2:** Definition of the parameters.

		Actual result	
		Positive	Negative
Expected result	Positive	TP	FN
	Negative	FP	TN

Acc_class_ is used to calculate the ratio between the number of correctly classified postural behavior and the total number of postural behaviors.

$${\text{Acc}}_{{{\text{class}}}} = \frac{{\text{The Number of postural behavior correctly classified}}}{{\text{The total number of postural behavior}}}$$

The Mean Intersection over Union (MIoU) is a standard measure of semantic segmentation, which is used to calculate the ratio of the intersection and union of the true value and the predicted value.

$${\text{MIoU}} = \frac{{{\text{TP}}}}{{{\text{FP}} + {\text{FN}} + {\text{TP}}}}$$

$${\text{MIoU}} = \frac{1}{n + 1}\mathop \sum \limits_{i = 0}^{n} \frac{{{\text{P}}_{ii} }}{{\mathop \sum \nolimits_{j = 0}^{n} {\text{P}}_{ij} + \mathop \sum \nolimits_{j = 0}^{n} {\text{P}}_{ji} - {\text{P}}_{ii} }}$$

where ${\text{P}}_{ii}$ indicates that the *i*-type is predicted as *i*-type, and ${\text{P}}_{ij}$ indicates that the *i*-type is predicted as *j*-type.

We set the weight based on the frequency of category *i* or *j*, and multiply it by the intersection over union (IoU) of each category, and sum into the frequency weighted intersection over union (FWIoU).

$${\text{FWIoU}} = \frac{{{\text{TP}} + {\text{FN}}}}{{{\text{TP}} + {\text{FP}} + {\text{TN}} + {\text{FN}}}} \times \frac{TP}{{{\text{TP}} + {\text{FP}} + {\text{FN}}}}$$

$${\text{FWIoU}} = \frac{1}{{\mathop \sum \nolimits_{i = 0}^{{\text{k}}} \mathop \sum \nolimits_{j = 0}^{{\text{k}}} {\text{P}}_{ij} }}\mathop \sum \limits_{i = 0}^{{\text{k}}} \frac{{\mathop \sum \nolimits_{j = 0}^{{\text{k}}} {\text{P}}_{ij} {\text{P}}_{ii} }}{{\mathop \sum \nolimits_{j = 0}^{{\text{k}}} {\text{P}}_{ij} + \mathop \sum \nolimits_{j = 0}^{{\text{k}}} {\text{P}}_{ji} - {\text{P}}_{ii} }}$$

## Image data processing

### Object extraction and classification

**Figure 2:**
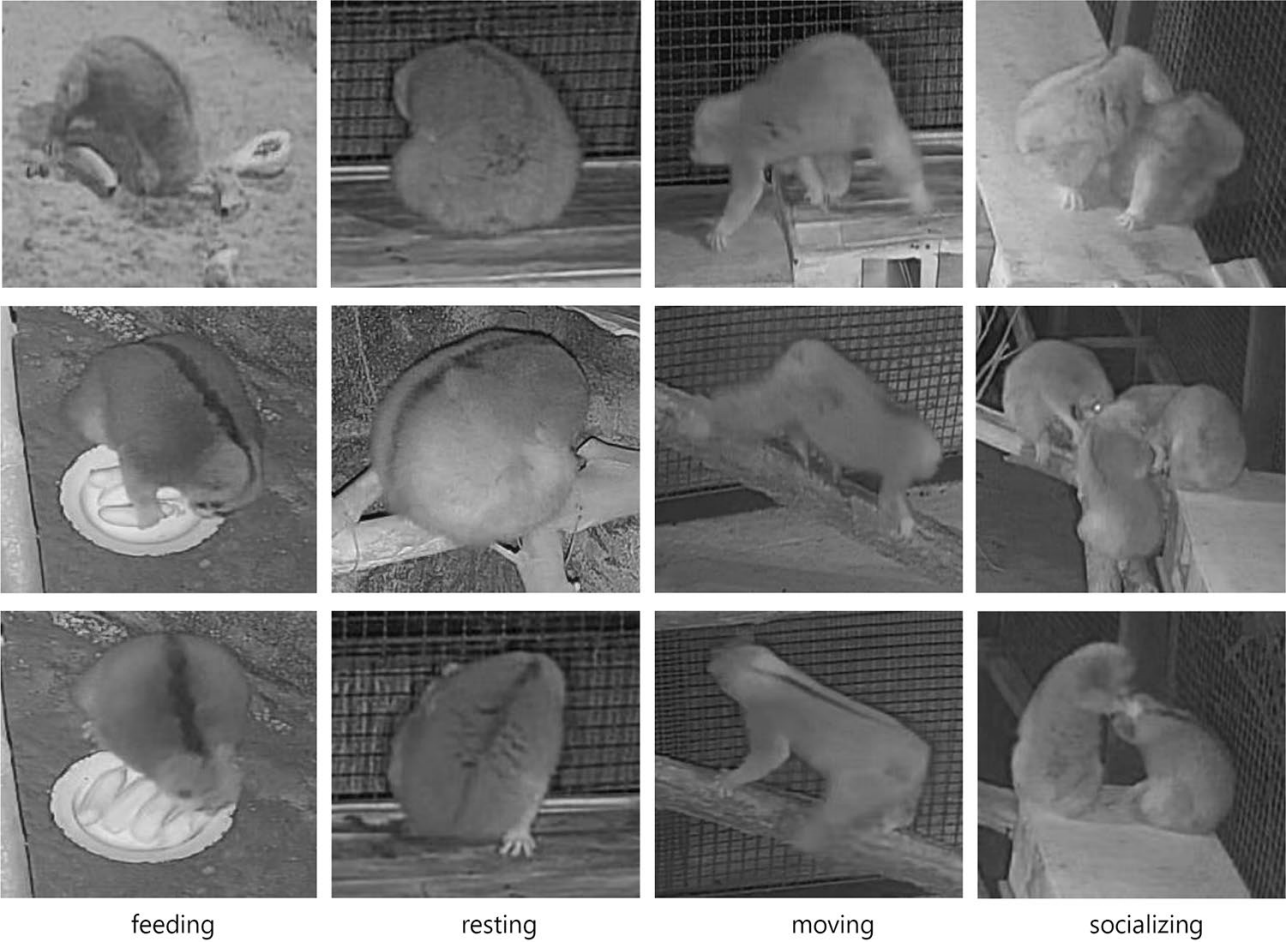
The images of postural behavior extracted by YOLOv5.

The deep learning technology in object detection is generally divided into two categories: one- and two-stage detector^35^. The one-stage detector is an end-to-end process which does not need to generate candidate frames. It directly converts the positioning problem of the object frame into a regression-processing problem. Based on the candidate area, the two-stage object-detection algorithm initially generates a series of candidate frames as samples, and then classifies them via the convolutional neural network (CNN). While the two-stage detection is represented by Faster R-CNN^36–38^, the YOLO series are the most representative algorithms in the one-stage object detection^39^. As the latest version in this series, YOLOv5 has made major advances in training speed and accuracy^40^. In present study, the YOLOv5 algorithm is used to extract the target individuals from the input image dataset (Fig. 2), and identify the three postural behavior types: *feeding*, *socializing*, and *move-rest*. Four networks (YOLOv5s, YOLOv5m, YOLOv5x and YOLOv5l) were generated by YOLOv5, and the YOLOv5s presented the superior performance due to a higher speed and accuracy rate. While the processing speed is acceptable, the highest accuracy rate reaches 95.1%.

### Feature extraction network used in semantic segmentation

#### ResNet

ResNet, derived from VGG19 network, is a convolutional neural network proposed by Microsoft Research^41^. Joined with the residual units, ResNet network effectively alleviate the gradient disappearance and model degradation by a short-circuit mechanism. In addition, the ResNet CNN uses jump connections and consequently alleviate the problem of the vanishing gradient caused by increasing depth in a deep neural network.

#### MobileNet

The MobileNet model is a lightweight deep neural network proposed by Google in 2017, and the MobileNet family includes MobileNetV1, MobileNetV2, and MobileNetV3^42^. The model is a simple streamlined architecture that replaces the regular convolution layer with depth wise separable convolution layer. MobileNets are low-latency and low-power models that yield small networks. It is one of the most commonly deployed models in edge computing due to limited parameters, reduced computation and high accuracy.

#### Xception

Xception is an extension of Inception V3 proposed by Google which replaces the standard Inception modules with deep separable convolutions^43,44^. The Xception architecture has 36 convolutional layers forming the feature extraction base of the network. The layers are structured into 14 modules, all of which have linear jump connections except for the first and last modules. Xception significantly outperforms Inception V3 due to a more efficient use of model parameters without increasing the complexity of the network^43^.

#### Dilated Residual Networks (DRN)

**Figure 3:**
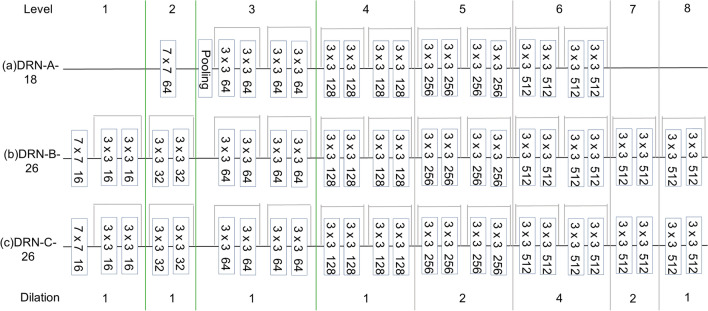
DRN network structure diagram.

By replacing the under-sampling layer inside the residual network model with dilated convolution, DRN (Dilated Residual Networks) yield higher accuracy in ImageNet classification than their non-dilated counterparts^45^, without increase in depth or model complexity. However, the use of dilated convolutions may lead to gridding artifacts. In this section, we develop a scheme for removing this effect from output activation maps produced by DRN (Fig. 3). An initial DRN constructed is referred to as DRN-A, which uses dilated convolution instead of under-sampling. We replace the pooling layers with convolution filters. An intermediate stage of the construction is referred to as DRN-B and the final construction is referred to as DRN-C.

### Contour extraction and classification of *moving* and *resting*

**Figure 4:**
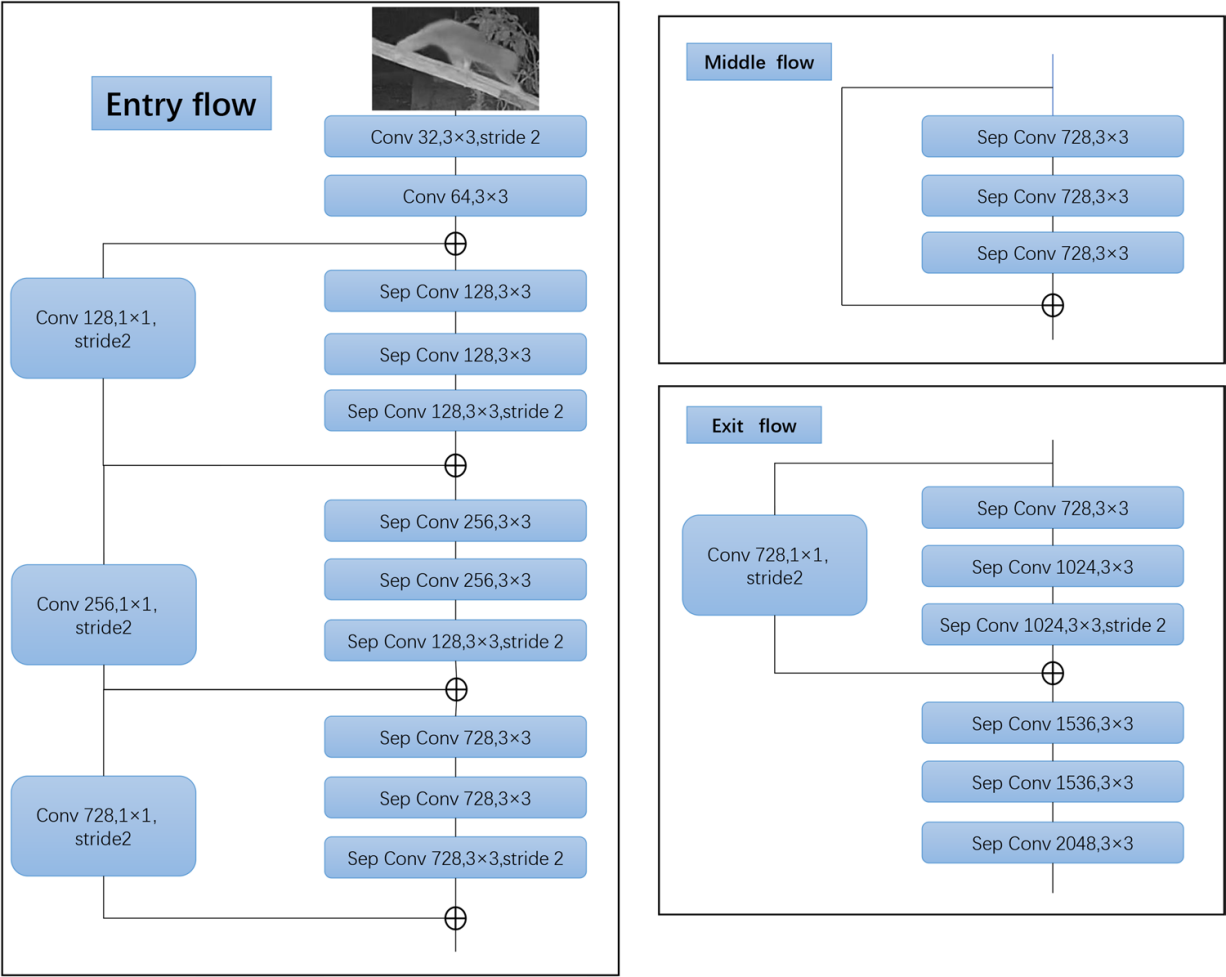
DeepLabv3 + network structure diagram.

**Figure 5:**
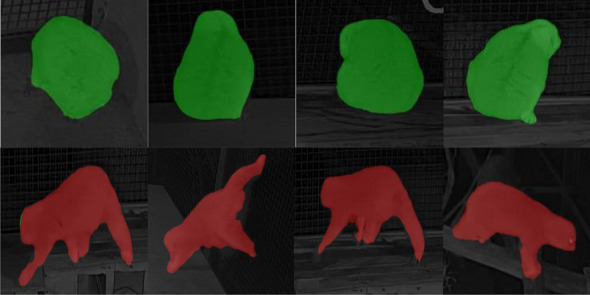
Semantic segmentation image of loris postural behavior (red indicate *moving* behavior and green indicate *resting*).

Semantic segmentation aims to assign a categorical label to every pixel in an image^46^. The DeepLab network, proposed by Google^47^, is specifically designed to deal with semantic segmentation, and four versions are currently released, namely DeepLabv1, DeepLabv2, DeepLabv3 and DeepLabv3 + .By adding a simple but effective decoder module, Deeplabv3 + extend to refine the segmentation results, particularly along object boundaries (Fig. 4). It further explores the Xception model and apply the depthwise separable convolution to Atrous Spatial Pyramid Pooling and decoder modules, leading to a faster and stronger network. As one of the most popular encoder-decoder networks, DeepLabv3 + include encoding and decoding paths. The encoder uses Dynamic CNN network (or Xception, VGG, ResNet) as backbone to extract basic features, and then uses dilated convolution to extract feature maps, and finally mix them with a 1 × 1 convolution. In the decoder part, the encoder features are first bilinearly upsampled and then concatenated with the corresponding low-level features from the network backbone. After the concatenation, a few 3 × 3 convolutions were applied to refine the features followed by another simple bilinear upsampling^48^. The effects of semantic segmentation are illustrated in Fig. 5.

### Model evaluation

**Figure 6:**
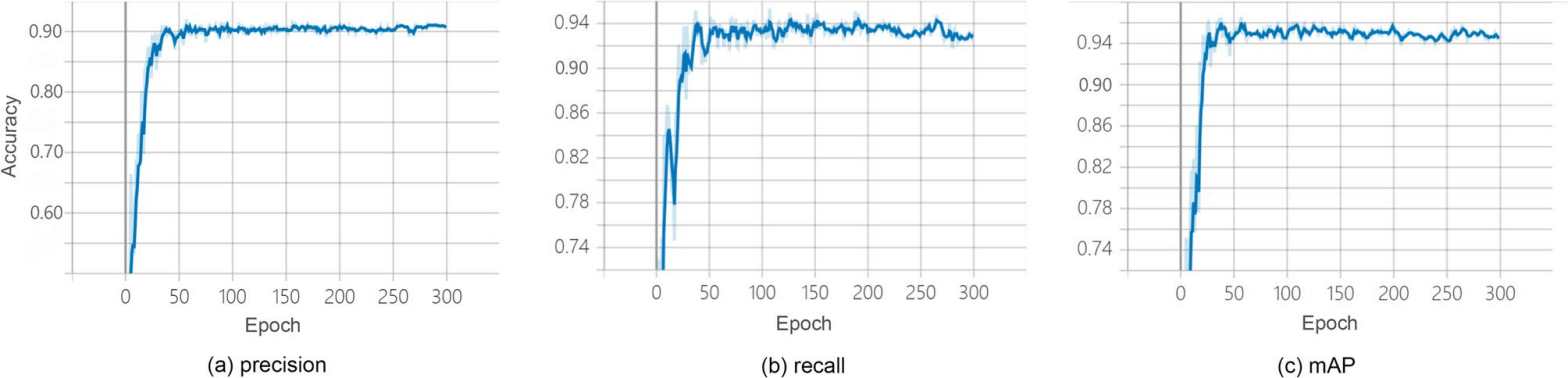
Precision, recall and mAP of object detection classification.

**Figure 7:**
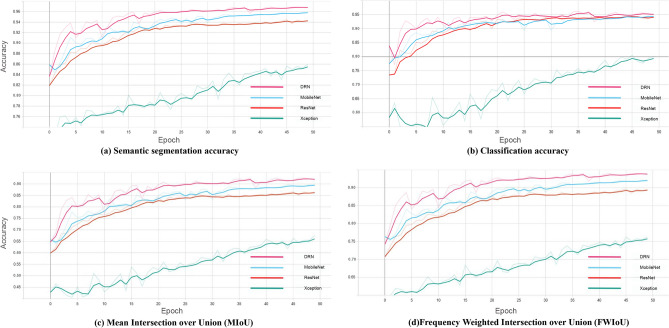
Evaluation index of the second step of joint training: (**a**) Semantic segmentation accuracy, (**b**) Classification accuracy, (**c**) MIoU, (**d**) FWIoU.

The precision, recall and mAP of the object detection were shown in Table 3 and Fig. 6, and the training effects of the semantic segmentation are shown in Table 4 and Fig. 7. The recognition accuracy of *socializing*, *feeding* and *move-rest* reached 95.1%. In the second step of joint training, the DRN feature extraction network showed the best performance. The accuracy of DRN-based semantic segmentation reached 96.8%, and the classification accuracy of *moving* and *resting* reached 95.2%.

**Table 3:** The effect of the YOLOv5 network.

Postural behavior	Precision	Recall	mAP
All	0.951	0.938	0.949
Feeding	0.951	0.932	0.951
Move-rest	0.941	0.948	0.953
Socializing	0.961	0.940	0.942

**Table 4:** Training effects of four different networks.

Network	Training time	Category	Eopch10	Eopch20	Eopch30	Eopch40	Epoch50
DRN	2h20m	Acc	0.926	0.955	0.961	0.964	0.968
		Acc_Class_	0.913	0.940	0.940	0.943	0.952
MobilNet	38 m	Acc	0.908	0.933	0.939	0.955	0.959
		Acc_Class_	0.902	0.916	0.894	0.939	0.943
ResNet	1h23m	Acc	0.895	0.928	0.933	0.938	0.944
		Acc_Class_	0.878	0.934	0.938	0.938	0.944
Xception	1h34m	Acc	0.764	0.782	0.815	0.847	0.860
		Acc_Class_	0.582	0.677	0.706	0.787	0.801

The evaluation index shows that YOLOv5 has a high accuracy in object detection classification, while the DRN is superior to other feature extraction networks in semantic segmentation. The present study aims to detect a precise behavior recognition algorithm to pave the way to constant monitoring of nocturnal animals. In this way, the accuracy rate has priority over other indicators, such as training speed. Thus, the DRN is finally adopted as our backbone.

## Discussion

This study created a novel model for automatic postural behavior recognition of confiscated Bengal slow lorises. For this nocturnal primate species, the key frames were extracted from night-vision surveillance video, and a combined method of object detection and semantic segmentation was introduced. Compared with domestic and farm animals, the captive slow lorises have more flexible limbs and diverse locomotion postures, and the boundaries of the semantic segmentation images between each behavior are relatively blurred with few differences. Therefore, the unified classification using the traditional method of semantic segmentation is limited in its ability to recognize behaviors. Given the simplified and stable enclosure environment in captive settings, we took into account the postural behavior itself and the surrounding background in identification. The behavioral types with obvious background characteristics and action features were classified in initial object detection, and together with the semantic segmentation process, a relatively high recognition accuracy was achieved. In addition, image data was extracted from a frame every two seconds in realizing the real-time monitoring of loris postural behavior. Both high accuracy and processing speed imply the integrated approach of YOLOv5 and DeepLab v3 + is qualified in behavior recognition of confiscated slow lorises, and shows promise for application to other captive nocturnal animals.

Since our current data is collected by a limited number of surveillance cameras which are mostly located at the upper side of the cage, the observation angle is restricted in a certain area, leading to a disproportionate dominance of dorsal pictures of slow loris in the image dataset. Like other studies in video-based behavior recognition (e.g. ^13,49^), the restricted camera number, coverage and angle impede the efforts to obtain qualified images. In addition, in contrast to the diurnal counterparts, most of the night-vision images of nocturnal animals had lower resolution quality^50^, making the individual boundaries difficult to be identified. Therefore, in further study, multiple high-resolution surveillance cameras should be set up at different angles in the enclosures. Moreover, in the three captive sites of the present study, the slow lorises were mostly housed in a group. Feeding and resting behavior displayed by two or more individuals together may be recognized as socializing in automatic identification. The three-way decision rule can be introduced into the subsequent test, namely that one can make a delayed decision on the recognition when the behavior types were characterized by similar features^51^.

In recent decades, traditional CNN models have achieved dramatic progress on image recognition, and a large number of extensions to process video data have been proposed. However, these models have limited capabilities to process variable length of input sequences. Given that animal behavior is composed of consecutive events, the constant monitoring based on time series may be unfeasible under the current networks, and thus the welfare-related abnormal repetitive actions, e.g. stereotypical behavior, cannot be detected. As an alternative approach, Recurrent Neural Networks (RNN) inputs the hidden layer data of the previous moment as the data of the current moment, allowing the temporal information to be preserved^52^. Compared with the traditional algorithms which assume a fixed spatio-temporal receptive field, RNN can be compositional in spatial and temporal layers. To overcome the limitation of simple RNN models known as “vanishing gradient”, Long Short-Term Memory (LSTM) RNN model has been proposed further^53,54^. In this way, the LSTM-RNN would be a promising network to be involved in achieving the goals of automatic behavior detecting, recognizing and monitoring.

Computer vision has been emerging as a new tool in the real-time automation of animal monitoring systems due to its non-intrusive and non-invasive properties, as well as its ability to present high throughput information. While Precision Livestock Farming has become a reliable solution to the challenges in automatic monitoring of domestic animals and assessment of welfare status^55^, only a few models related to computer vision were provided for wild animals. For those living in captive or semi-captive settings, without a sensor or collar, video data-based deep learning technology appears to be a feasible approach in automatic behavior recognition and welfare evaluation. The present framework provided a reliable, objective and reproducible method in measuring slow loris behavior. While husbandry activities are usually scheduled for the convenience of caregivers^56^, the models also have the potential to overcome the time restrictions in manual observation by expanding the datasets at a 24/7 time scale, which is particularly important in meeting the needs of nocturnal animals. Unfortunately, the current framework is too limited to identify more detailed ethograms or postures of captive or semi-captive slow lorises. Thus, this attempt must be considered preliminary and a case study, and in future research, we will look into how an advanced computer vision technology would measure more complex physiological and ethological responses to husbandry conditions, and precisely distinguish normal, abnormal or disturbed behavior in a wide range of species.

## Conclusion

While computer vision has been increasingly used in farm animal monitoring, research on captive or semi-captive wild animals remains scarce, impeding the efforts to precisely evaluate their housing conditions and welfare status. We introduced the deep learning technology into the postural behavior recognition of a nocturnal primate species. An object detection + semantic segmentation network displayed high accuracy in classifying four behavior types. As a case study, we investigate the potential of deep learning technology for the behavior recognition and classification of the captive nocturnal primates. The results show that YOLOv5 and DeepLabv3 + based on DRN have acceptable processing speed and accuracy in preliminary posture recognition, and paired with other machine learning technology, the model would contribute to establish a wide range of dataset for behavior ecology analysis and welfare improvement of captive or semi-captive animals.
